# Mechanistic Insights Into hsa_circ_0005654‐Induced Ferroptosis in Diabetic Foot Ulcers Through IGF2BP2 Interaction

**DOI:** 10.1002/kjm2.70047

**Published:** 2025-06-12

**Authors:** Chun‐Meng Li, Xiang‐Jian Zheng, Shang‐Shang Xie, De‐Yong Lin, Zi‐Tian Liu

**Affiliations:** ^1^ Department of Vascular Surgery Wenzhou Central Hospital Wenzhou City China

**Keywords:** diabetic foot ulcers, ferroptosis, hsa_circ_0005654, IGF2BP2, inflammation

## Abstract

This study investigated the molecular mechanism by which hsa_circ_0005654 aggravates diabetic foot ulcers (DFUs) by promoting ferroptosis. A DFU rat model was established, and lentiviral vectors interfering with circ_0005654 and IGF2BP2 expression were injected into DFU rats by tail vein. The successful injection of lentiviral vectors was verified by RT‐qPCR. The histopathology of foot ulcer tissues of DFU rats was observed by HE staining. Iron deposition was observed by Perls' Blue staining. Inflammatory factors were detected by ELISA. Protein expression of ferroptosis markers (GPX4 and SLC7A11) was measured by Western blot. The interaction of circ_0005654 with IGF2BP2 was verified by RNA pull‐down assay and RIP assay. Iron deposition and inflammatory factor levels were increased in foot ulcer tissues of DFU rats, and wound healing was impaired. Liproxstatin‐1, a ferroptosis inhibitor, promoted wound healing in DFU rats by inhibiting ferroptosis and inflammation. circ_0005654 expression was up‐regulated. Down‐regulating circ_0005654 promoted wound healing in DFU rats by inhibiting ferroptosis and inflammation. circ_0005654 could interact with IGF2BP2, and up‐regulating IGF2BP2 attenuated the effects of circ_0005654 down‐regulation in DFU rats. circ_0005654 promotes ferroptosis to aggravate DFU by interacting with IGF2BP2.

## 1 Introduction

Modern society is plagued with diabetes, a metabolic disorder characterized by long‐term hyperglycemia, which can damage multiple organs [1]. Diabetic foot is a serious complication of diabetes caused by vascular occlusive disease [2]. Patients with diabetic foot ulcers (DFUs) are at higher risk of invasive infections, increasing their chances of amputation [3]. The rising global prevalence of diabetes has led to an increase in DFUs; therefore, developing more effective methods for detecting and treating DFUs is urgently needed.

Ferroptosis, a type of programmed cell death reliant on iron and not involving apoptosis, is marked by the build‐up of reactive oxygen species (ROS) and lipid peroxides within cells [4]. In the field of DFUs, ferroptosis has emerged as a hotspot for research. The presence of ferroptosis and ferritinophagy in DFUs is attributed to an excess of iron resulting from disorders in the iron metabolic pathway [5, 6, 7]. Persistent elevated blood glucose levels increase lipid peroxidation byproducts, ROS, and proteins linked to ferroptosis, possibly resulting in ongoing, non‐recovery DFUs [8]. TP53, a ferroptosis‐related gene, has been implicated in DFU pathogenesis and could serve as a potential biomarker [9]. Ferrostatin‐1, a ferroptosis inhibitor, reduces DFU inflammation and oxidative stress markers significantly [10] and prevents the formation of wounds [11]. However, more studies are required to pinpoint the precise molecular mechanism of ferroptosis in DFUs.

CircRNAs are endogenous noncoding RNAs with a closed loop instead of a 5' cap and 3' poly A tail [12]. There is no effect of RNA exonuclease on circRNAs, and their expression is more stable, which is proven in a variety of eukaryotic organisms [13]. The importance of circRNAs in the organism has been confirmed, and diabetes has been associated with abnormal expression of several circRNAs. circ_0000064, for example, stimulates diabetic nephropathy, tethered cell proliferation, and mesangial fibrosis [14]. Further, circ_001209 is known to exacerbate diabetic retinal vascular dysfunction [15]. In DFU, circ_0084443 can modify keratinocyte migration and proliferation [16].

This study analyzed high‐throughput sequencing data from five control samples and five DFU samples in the GSE114248 dataset, which showed upregulation of hsa_circ_0005654 in DFU. Therefore, this study aimed to investigate the molecular mechanism by which hsa_circ_0005654 aggravates DFUs by promoting ferroptosis.

## 2 Materials and Methods

### 2.1 Bioinformatics Analysis

Analysis was conducted on high‐throughput sequencing data from 5 control and 5 DFU samples in the GSE114248 dataset. The analysis of differentially expressed genes (DEGs) was conducted utilizing DESeq2 [17]. Screening for DEGs adhered to the edgeR filtering standards (log2(fold change) > 2, false discovery rate > 0.05) [18]. DEGs that were upregulated and downregulated were classified based on log2 (Fold Change) > 1 and log2 (Fold Change) < −1, respectively.

### 2.2 DFU Model Establishment

All animal experimental protocols were approved by the Ethical Committee for Animal Research of Wenzhou Central Hospital (No. 2022‐WZ415). The Guangdong Medical Laboratory Animal Center (Guangdong, China) provided 50 male Sprague–Dawley (SD) rats, each weighing between 180 and 220 g and aged between 4 and 6 weeks. Under controlled environments (22°C ± 2°C, 45% humidity, 12‐h light/dark cycle), the rats were reared with sterilized food. Diabetes was induced through a regimen of a high‐fat diet and the intraperitoneal administration of streptozotocin (STZ; 50 mg/kg; S0130, Sigma, St. Louis, MO, USA) over 5 days. The control group received a standard diet and an identical quantity of sodium citrate buffer. Rats with blood glucose levels higher than 250 mg/dL were chosen as diabetes models after a 7‐day period. Diabetic rats were anesthetized using isoflurane, and then rectangular incisions of 2 mm by 5 mm were made on the right instep surface to simulate DFUs.

### 2.3 Animal Treatment

RiboBio (Guangdong, China) engineered and produced lentivirus vectors (1 × 10^8^ UT/50 μL) containing circ_0005654 shRNA (sh‐circ_0005654), along with a short interfering RNA negative control (sh‐NC), IGF2BP2 overexpression plasmid (pcDNA‐IGF2BP2), and an overexpression negative control (pcDNA‐NC). Liproxstatin‐1 (Lip‐1; an inhibitor of ferroptosis; S7699, Selleck, Shanghai, China) underwent dilution using 0.01% DMSO in saline, achieving a concentration of 1 μmol/L. SD rats were randomly allocated to 8 groups (*n* = 6 per group): Sham; DFU; Vehicle; Lip‐1; sh‐NC; sh‐circ_0005654; sh‐circ_0005654 + oe‐NC; sh‐circ_0005654 + oe‐IGF2BP2. After establishing the DFU model, rats were injected with the above drugs or lentiviral vectors via tail vein. Four weeks after injection, rats were anesthetized with 3% pentobarbital sodium (50 mg/kg) and foot ulcer tissue was collected.

### 2.4 Hematoxylin and Eosin (HE) Staining

Tissues from rat foot ulcers were sectioned into 4 μm paraffin slices, dewaxed, and hydrated. Subsequently, the sections were colored in hematoxylin for 5 min, differentiated using 5% acetic acid for one minute, exposed to a reversion blue solution for 1–2 min, colored in eosin solution for one minute, dehydrated using ethanol, and then sealed with neutral gum.

### 2.5 Perls' Prussian Blue Staining

Iron deposition in foot ulcer tissues of DFU rats was detected by Perls' Blue staining (Solarbio, Beijing, China). Tissues from rat foot ulcers were stained with potassium ferrocyanide and nuclear fast red. Leica microscopy was used to investigate morphological changes and iron distribution (DM4B, Germany).

### 2.6 Immunohistochemistry (IHC)

Sections of DFU tissues underwent dewaxing using xylene for 30 min and dehydration using alcohols (95%, 80%, and 75%), and were then incubated in 3% H_2_O_2_ for half an hour (Beyotime, China) at 37°C. The samples were immersed in a 0.01 M citrate buffer, heated to 95°C for 20 min, and subsequently cooled to ambient temperature. Post a 5‐min room temperature sealing in Ultra V Block, the samples underwent overnight probing with the anti‐mouse monoclonal antibody GPX4 (1:200, Thermofisher, USA) at 4°C. The following day, the samples were examined using a secondary antibody (1:2000, Thermofisher) for half an hour. Next, the sections were treated with streptavidin–biotin–peroxidase complex for 30 min and stained with 3 mL of diaminobenzidine (DAB) for 7 min. The sections were subsequently counterstained with hematoxylin, sealed, and fixed with neutral resin, observed in 3 fields of view under a microscope (Leica, Wetzlar, Germany), and analyzed using Image J software.

### 2.7 ELISA

The foot ulcer tissue homogenate was centrifuged, and the supernatant was collected. IL‐1β, TNF‐α and IL‐6 were quantitatively detected using the detection kits (Nanjing Jiancheng Bioengineering Institute).

### 2.8 RT‐qPCR

Using TRIzol reagent (Invitrogen, CA, USA), total RNA was isolated from rat foot ulcer tissues, followed by cDNA synthesis with a qRT‐PCR kit (Invitrogen). The RT‐qPCR process utilized a Stratagene RT‐PCR system (Applied Biosystems, USA), employing SYBR Green PCR Master Mix (DBI Bioscience). The protocol for thermal cycling included: a 2‐min session at 95°C, then 40 repetitions of 94°C for 20 s, 58°C for 20 s, and 72°C for 40 s. To measure circ_0005654 and IGF2BP2, GAPDH served as the internal standard using the 2^−ΔCT^ technique. Table 1 details the primer sequences.

**TABLE 1:** Primers.

Genes	Sequences (5′–3′)
circ_0005654	Forward: TTCAGTGCACAGCGAAAAGC
	Reverse: TCCTGGTGTCTTTTCAGGGC
IGF2BP2	Forward: GTTGGTGCCATCATCGGAAAGG
	Reverse: TGGATGGTGACAGGCTTCTCTG
GAPDH	Forward: GTCGGTGTGAACGGATTTG
	Reverse: TCCCATTCTCAGCCTTGAC

*Note*: GAPDH, glyceraldehyde 3‐phosphate dehydrogenase; IGF2BP2, insulin‐like growth factor 2 mRNA binding protein 2.

### 2.9 Western Blot

Lysis of the foot tissues was achieved through a radioimmunoprecipitation assay (RIPA) buffer (Solarbio), which included a protein phosphatase inhibitor. The proteins underwent separation through SDS‐PAGE and were then moved onto polyvinylidene difluoride membranes. Membranes underwent blocking in a mixture of Tris‐buffered saline and 0.1% Tween with 5% skimmed milk for an hour at ambient temperature, followed by an overnight incubation at 4°C using primary antibodies targeting glutathione peroxidase 4 (GPX4; 1:1000; Abcam, UK), solute carrier family 7 member (SLC7A11; 1:1000; Abcam), IGF2BP2 (1:5000; Proteintech, Shanghai, China), and GAPDH (1:5000; Proteintech). Subsequently, the membranes underwent a 1‐h incubation with a horseradish peroxide‐tagged anti‐IgG antibody (1:1000) matching the isotype, followed by the development of the blots using chemiluminescence reagent (Advansta, CA, USA). Visualization and quantification of the bands were performed using ImageJ software version 1.49.

### 2.10 RNA Pull‐Down

To confirm the binding relationship between circ_0005654 and IGF2BP2, an RNA traction test was carried out. The technique involved introducing biotinylated wild‐type circ_0005654 (Bio‐circ_0005654‐WT), mutant circ_0005654 (Bio‐circ_0005654‐MUT), and a negative control (Bio‐NC) into HEK293T cells (ATCC, USA). Following a 48‐h period, the cells were lysed, and subsequently, the gathered supernatant was subjected to a 3‐h incubation at 4°C with streptavidin magnetic beads (Invitrogen). Western blot analysis was carried out on purified RNA‐protein complexes.

### 2.11 RNA Binding Protein Immunoprecipitation (RIP) Assay

Using the IEMed‐K303 RIP kit (IEMed, Guangzhou, China), RIP detection was carried out. Lysis buffer was used to lyse the cells, and IgG or an anti‐IGF2BP2 antibody was then added and left overnight. After adding magnetic beads, proteinase K was applied to resuspended HEK293T cells in order to extract proteins. RNA was purified and analyzed by RT‐qPCR.

### 2.12 Statistical Analysis

Using SPSS 20.0, all continuous data were analyzed and presented as mean ± standard deviation. To assess significant differences, a one‐way analysis of variance was performed, and Tukey post‐test followed. The Student *t*‐test was performed for two‐group comparisons. A *p* value of less than 0.05 was deemed statistically noteworthy.

## 3 Results

### 3.1 Increased Iron Deposition and Inflammatory Factors in Foot Ulcer Tissues and Weakened Wound Healing Ability in DFU Rats

**FIGURE 1:**
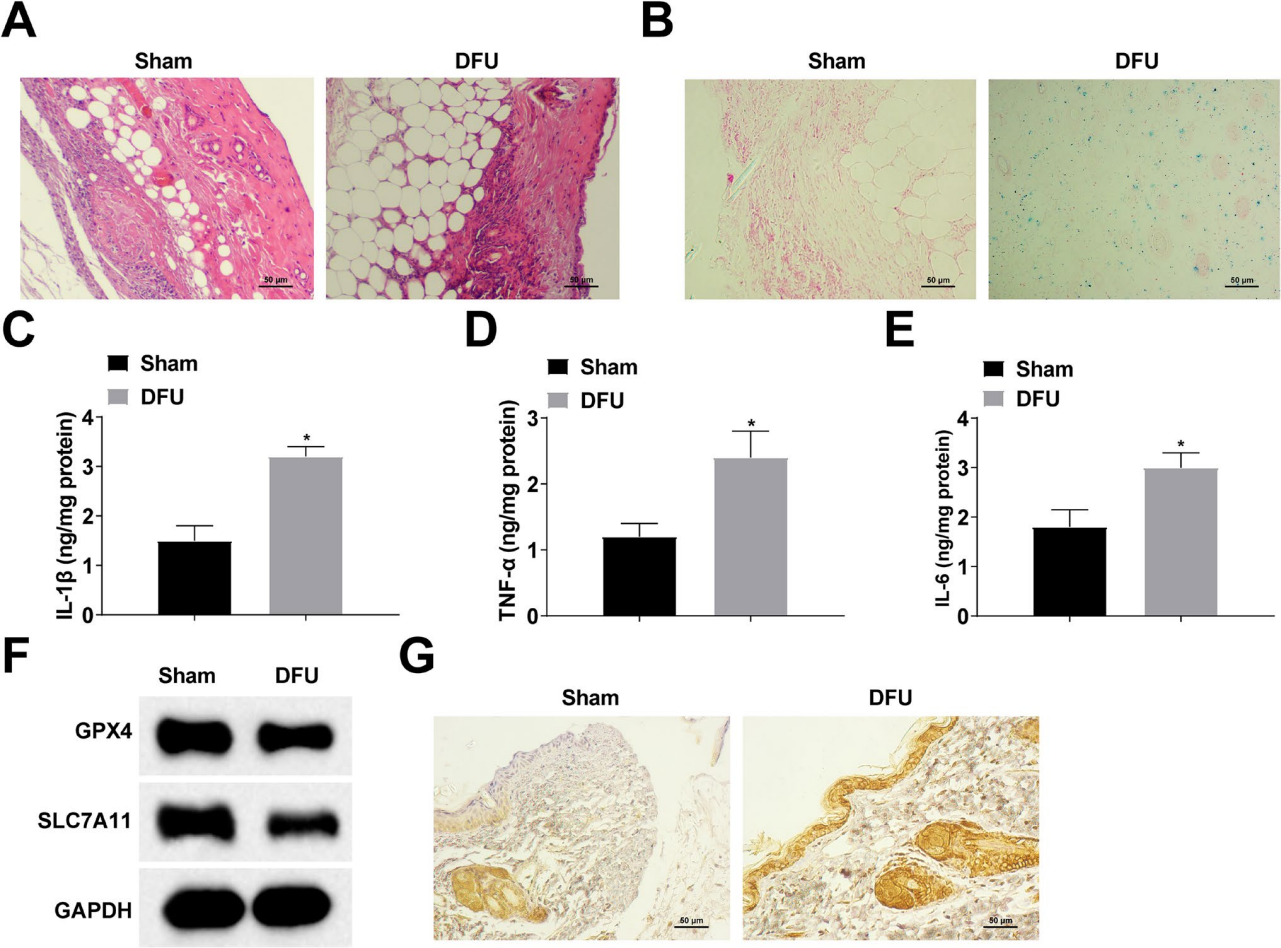
Increased intracellular iron deposition and inflammatory factors in foot ulcer tissues of DFU rats with reduced wound healing ability. (A) HE staining; (B) Perls' Blue staining; (C–E) ELISA for inflammatory factors (IL‐1β, TNF‐α and IL‐6) content; (F) Western blot for intracellular ferroptosis markers (GPX4 and SLC7A11) protein expression; (G) Immunohistochemical staining to detect GPX4 expression. *n* = 6. *p < 0.05 compared with Sham group.

A DFU rat model was established, and the histopathology of foot ulcer tissues was observed by HE staining, which showed that the foot ulcer tissues of DFU rats showed obvious inflammatory cell infiltration, and the wound healing ability was weakened (Figure 1A). Intracellular iron deposition in the foot ulcer tissues of DFU rats was observed by Perls' Blue staining, and the results showed that intracellular iron deposition in the foot ulcer tissues of DFU rats was significantly increased (Figure 1B). Inflammatory factors (IL‐1β, TNF‐α and IL‐6) in the foot ulcer tissues of DFU rats were detected by ELISA, and the results manifested a significant increase in the content of inflammatory factors in the foot ulcer tissues of DFU rats (Figure 1C–E). Intracellular ferroptosis markers (GPX4 and SLC7A11) were detected by Western blot. GPX4 and SLC7A11 were shown to be downregulated in the foot ulcer tissues of DFU rats (Figure 1F). In addition, immunohistochemical staining showed decreased GPX4 expression in foot ulcer tissues of DFU rats (Figure 1G).

### 3.2 Lip‐1 Promotes Wound Healing in DFU Rats by Inhibiting Ferroptosis and Inflammation

**FIGURE 2:**
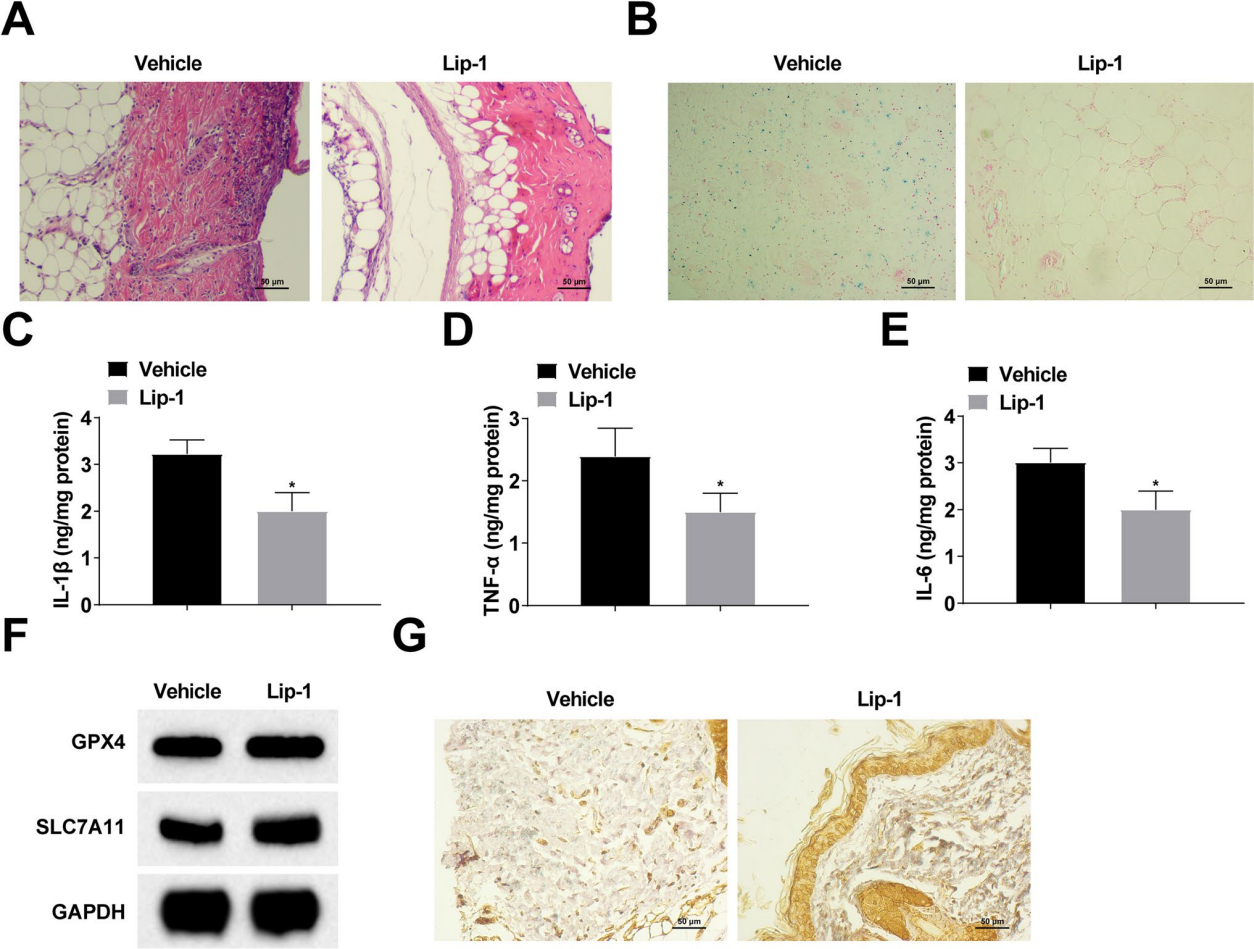
Lip‐1 promotes wound healing in DFU rats by inhibiting ferroptosis and inflammation. (A) HE staining; (B) Perls' Blue staining; (C–E) ELISA for inflammatory factors (IL‐1β, TNF‐α and IL‐6) content; (F) Western blot for intracellular ferroptosis markers (GPX4 and SLC7A11) protein expression; G: Immunohistochemical staining to detect GPX4 expression. *n* = 6. *p < 0.05 compared with Vehicle group.

To investigate the mechanism of ferroptosis in DFU, we injected 1 μmol/L Lip‐1 into the tail vein of DFU rats. HE staining demonstrated that inflammatory cell infiltration in foot ulcer tissues of DFU rats was reduced after Lip‐1 injection and showed better granulation formation (Figure 2A). Perls' Blue staining results showed that Lip‐1 could reduce intracellular iron deposition in foot ulcer tissues of DFU rats (Figure 2B). ELISA results revealed that Lip‐1 could reduce IL‐1β, TNF‐α, and IL‐6 content in foot ulcer tissues of DFU rats (Figure 2C–E). Western blot results showed that Lip‐1 could increase the GPX4 and SLC7A11 protein expression in foot ulcer tissues of DFU rats (Figure 2F). Immunohistochemical staining showed that Lip‐1 promoted GPX4 expression in foot ulcer tissues of DFU rats (Figure 2G).

### 3.3 circ_0005654 Downregulation Promotes Wound Healing in DFU Rats by Inhibiting Ferroptosis and Inflammation

**FIGURE 3:**
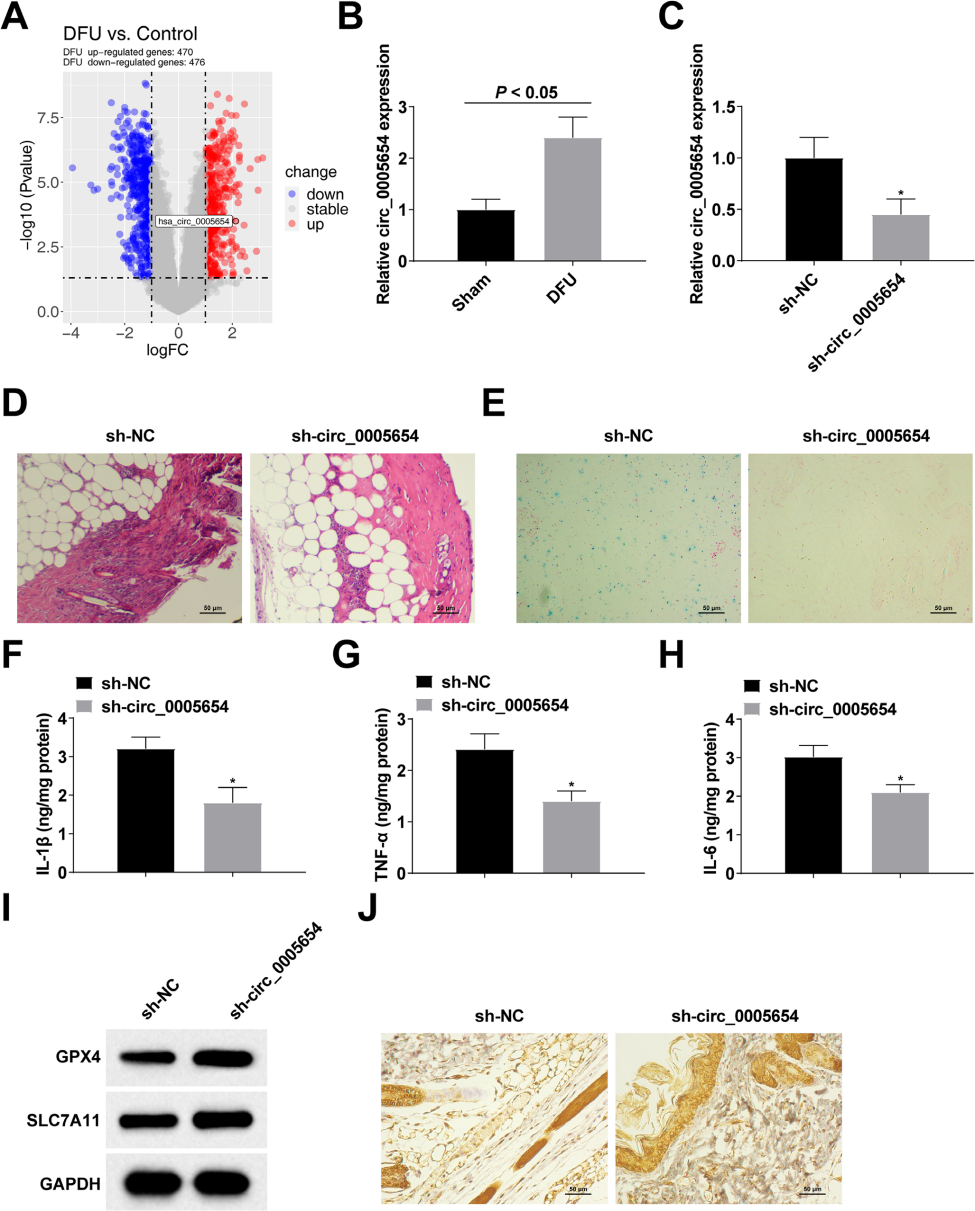
Down‐regulation of circ_0005654 promotes wound healing in DFU rats by inhibiting ferroptosis and inflammation. (A) Results of high‐throughput sequencing data analysis; (B, C) RT‐qPCR to detect circ_0005654 expression; (D) HE staining; (E) Perls' Blue staining; (F–H) ELISA to detect the content of inflammatory factors (IL‐1β, TNF‐α and IL‐6); (I) Western blot to detect protein expression of intracellular ferroptosis markers (GPX4 and SLC7A11); (J) Immunohistochemical staining to detect GPX4 expression. *n* = 6. *p < 0.05 compared with sh‐NC group.

The regulation of ferroptosis is thought to be controlled by noncoding RNAs [19, 20]. High‐throughput sequencing data of five control samples and five DFU samples in the GSE114248 dataset detected that circ_0005654 expression was significantly upregulated in DFU (Figure 3A). This upregulation trend was observed in DFU rats (Figure 3B). Therefore, we further explored the effect of circ_0005654 on DFU rats. The sh‐NC, sh‐circ_0005654 lentiviral vectors were injected into DFU rats by tail vein, and the successful injection was verified by RT‐qPCR (Figure 3C). Silencing circ_0005654 reduced inflammatory cell infiltration in foot ulcer tissues of DFU rats and exhibited better granulation formation (Figure 3D), reduced intracellular iron deposition (Figure 3E), reduced IL‐1β, TNF‐α and IL‐6 (Figure 3F–H), and increased protein levels of GPX4 and SLC7A11 (Figure 3I). Immunohistochemical staining showed that down‐regulation of circ_0005654 promoted GPX4 expression in foot ulcer tissues of DFU rats (Figure 3J).

### 3.4 circ_0005654 Can Interact With IGF2BP2

**FIGURE 4:**
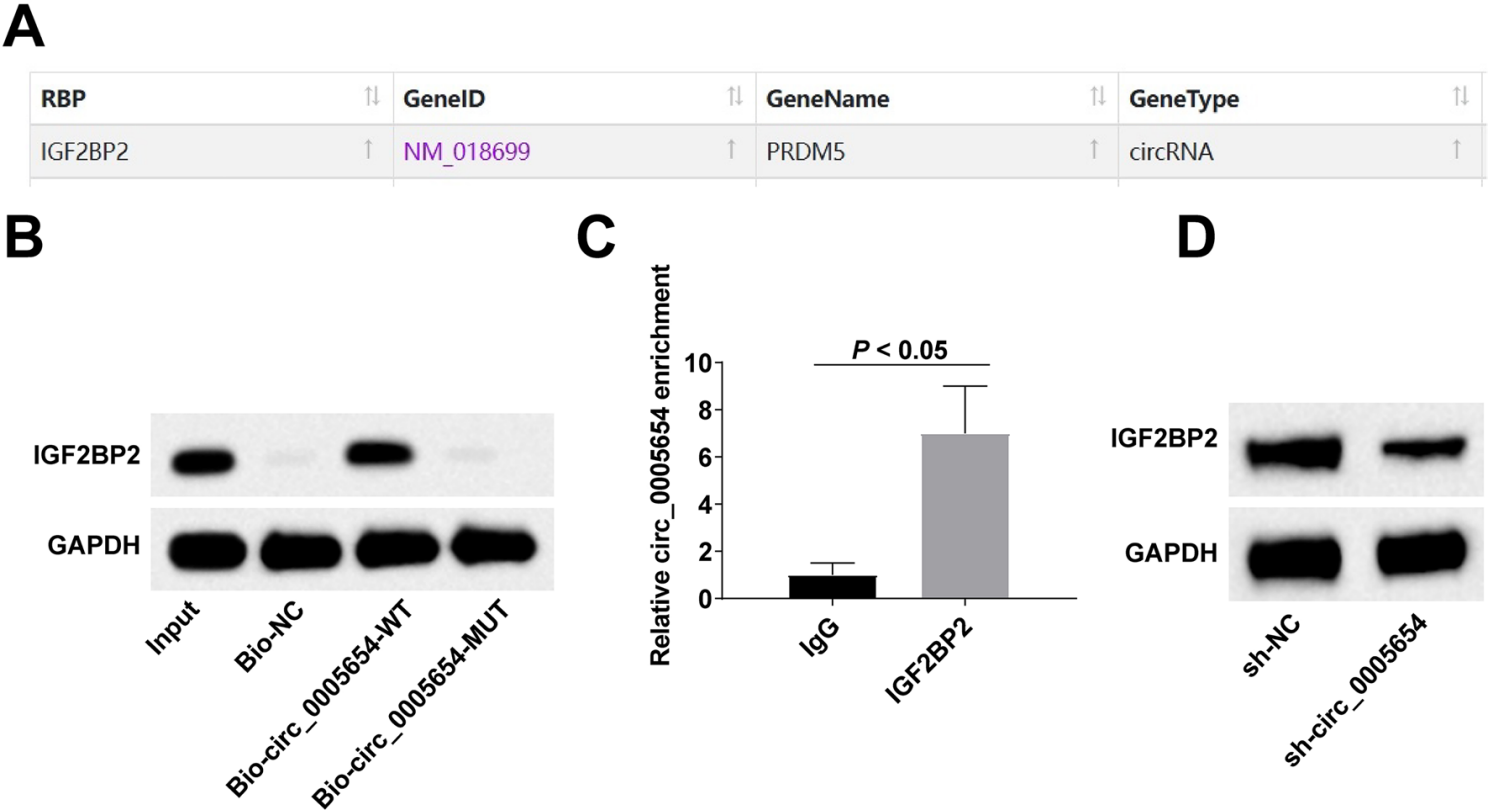
circ_0005654 can interact with IGF2BP2. (A) Bioinformatics website starBase analysis results; (B) RNA pull‐down experiment (*N* = 3); (C) RIP experiment (*N* = 3); (D) RT‐qPCR and Western blot to detect IGF2BP2 protein expression (*n* = 6).

Interactions between circRNAs and RNA‐binding proteins (RBPs) play a key role in their biological functions [21]. Analysis of the bioinformatics website starBase (https://starbase.sysu.edu.cn/) revealed an interaction between circ_0005654 and IGF2BP2 (Figure 4A). RNA pull‐down experiments showed that Bio‐circ_0005654‐WT could enrich the IGF2BP2 protein (Figure 4B). The relationship between endogenous IGF2BP2 and circ_0005654 was then verified by using the RIP test to probe the immunoprecipitates with an anti‐IGF2BP2 antibody (Figure 4C). Additionally, down‐regulating circ_0005654 was detected to inhibit IGF2BP2 expression (Figure 4D).

### 3.5 IGF2BP2 Overexpression Attenuates the Effect of circ_0005654 Downregulation in DFU Rats

**FIGURE 5:**
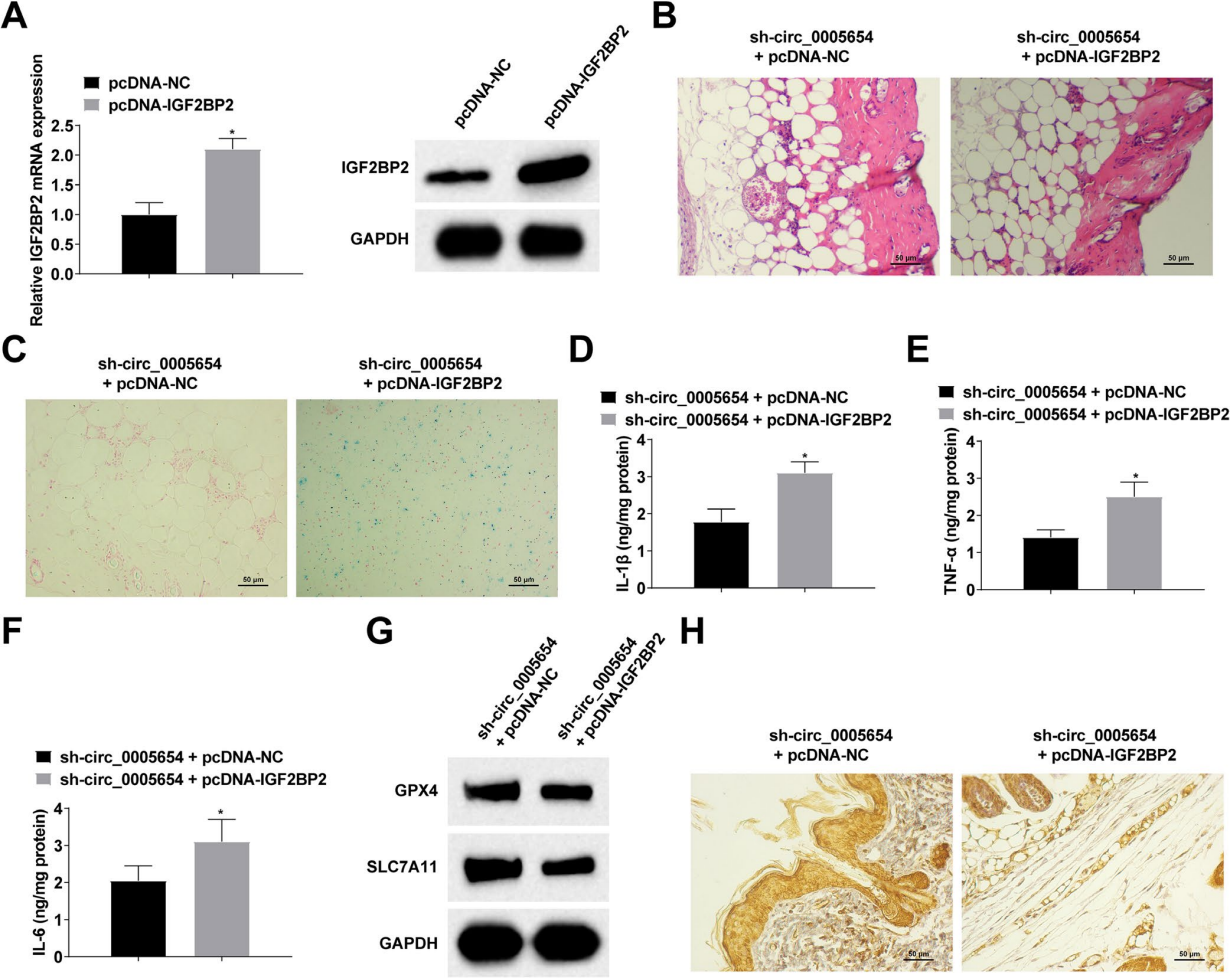
Down‐regulation of IGF2BP2 attenuates the effect of down‐regulation of circ_0005654 on DFU rats. (A): RT‐qPCR and Western blot to detect IGF2BP2 protein expression; (B) HE staining; (C) Perls' Blue staining; (D–F) ELISA to detect the content of inflammatory factors (IL‐1β, TNF‐α and IL‐6); (G) Western blot to detect the cellular protein expression of intracellular ferroptosis markers (GPX4 and SLC7A11); (H) Immunohistochemical staining to detect GPX4 expression. *n* = 6. *p < 0.05 compared with sh‐circ_0005654 + pcDNA‐NC group.

The sh‐circ_0005654 + pcDNA‐NC or sh‐circ_0005654 + pcDNA‐IGF2BP2 lentiviral vectors were injected into the tail vein of DFU rats, and the success of the injections was verified by RT‐qPCR and Western blot (Figure 5A). Various results showed that up‐regulating IGF2BP2 attenuated the effect of circ_0005654 downregulation on DFU rats (Figure 5B–H).

## 4 Discussion

DFU is pathophysiologically caused by microvascular dysfunction and peripheral neuropathy. Drugs that enhance nerve perception, improve microcirculation, and promote skin regeneration can be used to treat DFU, as well as anti‐inflammatory and anti‐infection medicines [22, 23, 24]. Patients with diabetes have been shown to experience iron overload [25, 26]. A pathological change such as this promotes insulin resistance and worsens diabetes‐related cardiovascular complications, as well as fosters oxidative stress [27].

The association between ferroptosis and DFU is suggested, yet few research efforts have focused on this relationship. One way that ferroptosis contributes to DFU is by the activation of inflammatory pathways. One of the main characteristics of DFU is chronic inflammation, which can cause tissue damage and poor wound healing [28]. In DFU, iron‐dependent lipid peroxidation stimulates the creation of pro‐inflammatory cytokines and chemokines, which enhance the inflammatory response. Furthermore, iron accumulation in tissues can enhance the process of ferroptosis as a result of lipid peroxidation. Ferroptosis may also be linked to peripheral neuropathy, which can result in loss of sensation in the feet, followed by foot ulcers [29]. Moreover, ferroptosis in animal models induces axonal degeneration and impairs nerve function [30, 31]. The mechanisms by which ferroptosis causes tissue damage and inflammation in DFU are not yet clear. In the present study, intracellular iron deposition and inflammatory factor levels were increased in foot ulcer tissues of DFU rats, and wound healing was impaired. The ferroptosis inhibitor Lip‐1 promoted wound healing in DFU rats by inhibiting ferroptosis and inflammation.

Non‐coding RNAs significantly influence diabetic wound healing through the regulation of ferroptosis [32, 33]. In this study, we analyzed the high‐throughput sequencing data of 5 control samples and 5 DFU samples in the GSE114248 dataset and found that circ_0005654 expression was upregulated in DFU. Additionally, circ_0005654 expression was similarly upregulated in DFU rats. Furthermore, reducing circ_0005654 promoted wound healing in DFU rats by reducing ferroptosis and inflammation.

It appears that circRNAs may play an essential role in human diseases, acting as miRNA sponges or interfering with RBP [34]. It has been shown that some non‐coding RNAs regulate IGF2BP2 expression by binding to and regulating IGF2BP2 [35, 36]. In this study, IGF2BP2 was the RBP of circ_0005654 and was positively regulated by circ_0005654. In addition, IGF2BP2 overexpression attenuated the effect of circ_0005654 down‐regulation in DFU rats.

However, there are limitations of this study. First, we only performed animal experiments, and we need to further validate our findings in cellular experiments in the future. Additional studies are required to ascertain if circ_0005654 influences DFU through alternative mechanisms, which could provide new insights into DFU pathophysiology.

## 5 Conclusions

circ_0005654 exacerbates DFUs by interacting with IGF2BP2 and positively regulating IGF2BP2 expression to promote ferroptosis and inflammation. The circ_0005654/IGF2BP2/ferroptosis axis is involved in DFU wound healing, thus providing a new therapeutic target.

## Ethics Statement

All animal experiments were compliant with the ARRIVE guidelines and performed in accordance with the National Institutes of Health Guide for the Care and Use of Laboratory Animals. The experiments were approved by the Institutional Animal Care and Use Committee of Wenzhou Central Hospital (No. 2022‐WZ415).

## Conflicts of Interest

The authors declare no conflicts of interest.

## Data Availability

The data that support the findings of this study are available from the corresponding author upon reasonable request.
